# Entomopathogenic nematode management of small hive beetles (*Aethina tumida*) in three native Alabama soils under low moisture conditions

**DOI:** 10.21307/jofnem-2021-063

**Published:** 2021-07-08

**Authors:** WinDi Sanchez, David Shapiro, Geoff Williams, Kathy Lawrence

**Affiliations:** 1 Department of Entomology and Plant Pathology, Auburn University, Auburn, AL, 36849; 2 209 USDA-ARS, SEA, SE Fruit and Tree Nut Research Unit, 21 Dunbar Road Byron, GA, 31008; 3 Department of Entomology and Plant Pathology, 301 Funchess Hall, Auburn University, Auburn, AL, 36849; 4 Department of Entomology and Plant Pathology, 559 Devall Dr., CASIC Building, Auburn University, Auburn, AL, 36849

**Keywords:** Biological control, Entomopathogenic nematode, *Heterorhabditis bacteriophora*, *H. indica*, *Steinernema carpocapsae*, *S. feltiae*, *S. kraussei*, and *S. riobrave*

## Abstract

The goal was to determine the efficacy of entomopathogenic nematodes (EPNs) on *Aethina tumida* small hive beetle (SHB) in Alabama soils. The objectives were to (i) determine the pupation success of SHB wandering larvae; (ii) determine the efficacy of EPNs on SHB wandering larvae in natural and autoclaved soil; and (iii) determine the efficacy of EPNs on SHB wandering larvae in three Alabama soil types at typical low moisture levels. The Alabama soils were Kalmia loamy sand (KLS), Benndale fine sandy loam (BFSL), and Decatur silt loam (DSL). *Heterorhabditis bacteriophora*, *H. indica, Steinernema carpocapsae, S. feltiae*, S. *kraussei*, and *S. riobrave* were tested at population densities of 5, 10, 20, 40, and 80 third-stage infective EPN juveniles (IJ3) per 130 cm^3^ soil. Pupation success in SHB population densities of 5, 10, and 20 wandering larvae per Petri dish were similar. Of the six EPN species, *S. carpocapsae* achieved the highest efficacy across all EPN population densities in both natural and autoclaved soil. *Steinernema riobrave* and *H. indica* achieved the next highest efficacies; however, they were significantly less effective than *S. carpocapsae. Steinernema carpocapsae* parasitized 87% SHB wandering larvae across all population densities tested. *Steinernema carpocapsae* achieved the best efficacy colonizing 94% of the SHB in the KLS soil, 80% in the BFSL soil, and 47% in the DSL soil. In conclusions, *S. carpocapsae* is be a promising biological control EPN to implement into a management system on SHB.

*Apis mellifera* (Linnaeus, 1758) honey bees are pollinators that support crop growth and environmental health globally. Their pollination services, along with sales of hive products, makes *A. mellifera* management a billion-dollar industry (Mortensen et al., 2013; Smith et al., 2013). Pests of *A. mellifera* colonies can negatively affect overall colony health, hive products, and productivity. One such pest is *Aethina tumida* (Murray, 1867) (Coleoptera:Nitidulidae) small hive beetle (SHB), a secondary opportunistic pest that completes most of its lifecycle inside bee colonies and pupates in the soil under or around colonies (Willcox et al., 2017; Zawislak, 2014). Infestation of an *A. mellifera* colony begins with adult *A. tumida* flying to a suitable hive, mating, and laying eggs in clusters within the wax frames (Ellis, 2004; Graham et al., 2011; Mustafa et al., 2015; Neumann and Elzen, 2004). The larvae develop by consuming pollen, honey, and *A. mellifera* brood, consequently damaging the frames, fermenting honey, and creating suitable conditions for other pests to thrive (Ellis et al., 2002; Neumann et al., 2013). Larval development speed is dependent on food availability and temperature (Neumann et al., 2013, 2016). When the larvae are ready to pupate, they drop beneath the hive and begin searching for a suitable pupation location. At this stage, they are called wandering larvae. Once in the soil, *A. tumida* develop into pupae and emerge as adults 13 to 74 days later depending on temperature and soil moisture levels (Neumann et al., 2013).

Control measures for *A. tumida* have included maintaining strong *A. mellifera* colonies, breeding *A. mellifera* for hygienic behavior, monitoring hives for adults and larvae, removing damaged frames, purchasing and baiting *A. tumida* traps, and chemical treatments in or around infested hives (Cuthbertson et al., 2013; de Guzman et al., 2001; Ellis, 2012; Ellis et al., 2003, 2004a; Neumann et al., 2016; Smith et al., 2013; Zawislak, 2014). Integrated Pest Management (IPM) systems are currently the best option for SHB control because chemical applications can affect *A. mellifera* individuals as well as hive products (Berry et al., 2013; Fulton et al., 2019).

Recent laboratory bioassay studies suggest that entomopathogenic nematodes (EPNs) can successfully infect, feed on, and reproduce in *A. tumida* wandering larva and pupa (Cabanillas and Elzen, 2006; Cuthbertson et al., 2012; Ellis et al., 2010; Hill et al., 2016; Shapiro-Ilan et al., 2010). Subsequently, these EPNs may represent an efficient biological control option for an IPM program. EPNs naturally live in soil and require an insect host to reproduce. The two main genera of EPNs that have been marketed for control of *A. tumida* in Europe and North America are *Steinernema* spp. and *Heterorhabditis* spp. These EPN genera have different hunting styles and each species have different environmental and host preferences (Shapiro-Ilan et al., 2002). *Steinernema* spp. generally hunt insect hosts using ambush (sit and wait) techniques while *Heterorhabditis* spp. hunt using cruising (seek and attack) techniques (Ellis et al., 2010; Lewis et al., 1992; Wilson et al., 2012).

Six EPN species that have shown promise in laboratory bioassays for controlling *A. tumida* are *Heterorhabditis bacteriophora* (Poinar, 1975) *Heterorhabditis indica* (Poinar et al., 1992) *Steinernema carpocapsae* (Weiser, 1955), *Steinernema feltiae* (Filipjev, 1934), *Steinernema kraussei* (Steiner, 1923), and *Steinernema riobrave* (Cabanillas et al., 1994) (Cabanillas and Elzen, 2006; Cuthbertson et al., 2012; Ellis et al., 2010; Hill et al., 2016; Shapiro-Ilan et al., 2010). Infection of *A. tumida* wandering larva occurs when the EPN IJ3 enters the host through a natural orifice and releases a symbiotic gram-negative bacterium that lives within the EPN into the insect hosts hemocoel (Akhurst, 1982; Adams and Nguyen, 2002; Boemare et al., 1996; Kaya and Gaugler, 1993; Stock, 2019). Each of the EPN species mentioned above have a different symbiotic bacterium species that is responsible for killing the host and creating a suitable environment for EPN reproduction (Boemare et al., 1993). The EPNs produce two to three generations within the host cadaver before the new IJ3s leave the cadaver in search of a new host (Smart, 1995).

One limitation of EPNs as biological control agents is the effect of abiotic soil factors on efficacy. Soil particle size, available moisture, temperature, salinity, organic material content, and pH have all been found to affect EPN efficacy to varying degrees (Divya and Sakar, 2009; Kung et al., 1990a, 1990b, 1991; Kaspi et al., 2010; Koppenhöfer and Fuzy, 2006; Koppenhöfer et al., 1995; Molyneux and Bedding, 1984; Shapiro-Ilan et al., 2011; Tofangsazi et al., 2012). Furthermore, soil conditions have also been shown to affect *A. tumida* pupation success (Ellis et al., 2004b; Neumann et al., 2013, 2016). The majority of bioassays screening these EPNs for *A. tumida* management used soil that was sterilized by autoclaving because this removes all living biota from the soil and limits confounding variables within the study (Ellis et al., 2004b; Neumann et al., 2013, 2016). However, autoclaving the soil removes natural biotic competition from the soil and alters the physical and chemical properties so that conditions are not field-realistic. To date, there is little knowledge about how EPNs perform with natural competition in field soils with varing textures and limited available moisture. Introducing natural soil factors in laboratory bioassays is the next step toward field trials.

The main objective of this research is to determine the efficacy of EPNs to control *A. tumida* wandering larva in three soil types found within the state of Alabama at low moisture levels. Specifically the objectives were to (i) determine the pupation success of SHB wandering larvae in natural non-autoclaved and sterile autoclaved soil; (ii) determine the efficacy of EPNs on SHB wandering larvae in natural and autoclaved soil in low moisture conditions; and (iii) determine the efficacy of EPNs on SHB wandering larvae in three natural non-autoclaved soil types at low moisture levels. Adding EPNs to an IPM system for SHB may benefit beekeepers on a local, state, and national level, improve the health of *A. mellifera* colonies, and subsequent pollination rates in locations where SHB exist.

## Materials and methods

### Aethina tumida colony

Approximately 132 male and female SHB adults were field collected in September 2018 via a mouth-operated insect aspirator from ten active honey bee hives placed at the Auburn University (AU) Bee Lab, Auburn, AL. Infected *A. mellifera* colonies selected had not been used for chemical research. Adult SHB were sexed and placed into breeding jars based on protocol described in volume two of the COLOSS Beebook (Neumann et al., 2013). Each breeding jar was labeled and placed in incubators maintained at 25^o^C, 80% relative humidity (RH), in total darkness (Neumann et al., 2013). Adult and subsequent larvae in the breeding jars were provided a diet of 400 g Ultra Bee artificially bee pollen substitute purchased (Mann Lake, Hackensack, Minnesota) to consume and lay eggs on weekly (Neumann et al., 2013). Mature larvae were placed in plastic pupation jars filled with ~1.75 L sterilized soil that were placed in an incubatore at 25^o^C, 80% relative humidity (RH), in total darkness for 20 days (Neumann et al., 2013). Increasing genetic diversity in laboratory colonies is important to decrease the chances of inbreeding as well as decrease the potential for genetic branch between wild SHB and laboratory reared SHB. Genetic diversity was promoted in two ways. First, all wandering larvae were combined in a large bin before being placed into a pupation jar. Second, once emerged, adults were sexed and randomly placed into new breeding jars. Only the wandering larvae and pupae phase of the SHB lifecycle were utilized for the study.

### Entomopathogenic nematodes

Nematode genera were selected based on previous literature and current market availability. For this study, commercially purchased *Steinernema feltiae*, *Steinernema kraussei*, and *Heterorhabditis bacteriophora*, *Steinernema riobrave, Steinernema carpocapsae,* and *Heterorhabditis indica* third stage infective juveniles (IJ3) were tested (Arbico organics, Oro Valley, AZ). We also tested *S. riobrave, S. S. carpocapsae*, and *H. indica* IJ3 reared by the Dr. Shapiro-Ilan, USDA, in Byron, Georgia. EPNs were kept in a standard refrigerator at 4^o^C until they were needed for each experiment. All experiments were set up within 72 hr of EPN arrival. EPN IJ populations were prepared by placing a 75 µm mesh sieve on top of a 25 µm mesh sieve and running water indirectly through the sieves. Contents in the 25 µm sieve were collected into a glass beaker, diluted to 100 mL with water, and kept at room temperature. The sieves were triple rinsed thoroughly between each nematode species. Each EPN species were enumerated via a Nikon TSX 100 inverted microscope at 40 ×  magnification. For this study, we used five population density levels: 5, 10, 20, 40, and 80 IJ3/cm^2^ in 130 cm^3^ of soil following methods by Ellis et al. (2010) and Vega et al. (1994). These equated to approximately 120, 240, 485, 970, and 1940 IJ3s/1 mL inoculum.

### Soils

The soils used were collected from Auburn University research centers and consisted of a Kalmia loamy sand (KLS) (80% sand, 10% silt, 10% clay) from the Plant Breeding Unit in Tallassee, AL, a Benndale fine sandy loam (BFSL) (73% sand, 20% silt, 7% clay) collected from the Brewton Agricultural Research Unit in Brewton, AL, and a Decatur silt loam (DSL) (24% sand, 49% silt, 28% clay) from the Tennessee Valley Research and Extension Unit near Belle Mina, AL (Table 1). KLS represents the middle of the fine sand to heavy clay soil spectrum we tested to determine the efficacy of EPNs in the wide range of Alabama soils, therefore, KLS was used as the standard for all experiments. Each soil was kept in autoclave bags in a walk-in refrigerator set at 4^o^C until needed. Half of each of the soils was autoclaved three times at 121^o^C for 60 min with 24 hr between sterilizations (Bennett et al., 2003; Trevors, 1996; Wolf and Skipper, 1994). For the 24 hr between sterilizations, each bag was placed on a laboratory counter to cool to room temperature. After autoclaving, the soils were weighed and then placed in an oven at 38^o^C (Soil Survey Staff, 2011). Weight was checked every 24 hr until containers were no longer losing weight in accordance with Susha Lekshmi et al. (2014). The dry soil was placed in a new autoclave bag, sealed, and placed back into the walk-in cold room. Non-autoclaved soils were then weighed, placed into the oven at 38^o^C, and checked as described above. Once prepared, 130 cm^3^ of each soil was placed into a 25 mm × 100 mm Petri dish. The appropriate amount of moisture was mixed into each soil depending on objective before soils were placed back into the Petri dishes and weighed again. The second weight documented became the standard weight for each soil type.

**Table 1.** Chemical properties of non-autoclaved and autoclaved soils used in the study^a^.

		Non-autoclaved			Autoclaved		
Unit		DSL	BFSL	KLS	DSL	BFSL	KLS
ppm	Ca	22351	695	409	2085	376	230
	K	283	72	58	271	49	68
	Mg	258	62	174	228	147	72
	P	281	36	28	262	32	20
	Al	393	274	107	312	86	184
	B	1.2	0.4	0.4	1.2	0.4	0.4
	Cu	1.4	1	2.7	1.2	1.5	0.8
	Fe	7	22	26	12	19	21
	Mn	91	36	20	590	86	246
	Na	46	39	38	48	40	36
	Zn	20	4	2.9	15	2.4	2.9
	NO_3_-N	11	27	12	7	8	21
	CaCO_3_	3.8	<1.0	<1.0	2.5	<1.0	<1.0
	Soluble salts	238	238	1,428	381	159	254
mmhos/cm	Electrical conductivity	0.19	0.19	1.12	0.3	0.12	0.2
%	N	0.25	0.07	0.035	0.24	0.029	0.07
%	C	2.99	1.38	0.54	3.12	0.5	1.41
%	Organic Material	5.1	2.4	0.9	5.4	0.9	2.4
%	S	0.031	0.009	0.0073	0.027	0.0066	0.013
%	Moisture	0.57	0.93	0.026	0.89	0.26	0.68
cm^3^/cm^3^	H_2_O availability	0.16	0.09	0.08	0.16	0.07	0.09
	pH	6.00	4.59	6.94	7.00	6.85	5.29

Note: ^a^Soil types include Kalmia loamy sand (KLS) collected from AU Plant Breeding Unit in Tallassee, AL, Benndale fine sandy loam (BFSL) collected from AU Brewton Agricultural Research Unit in Brewton, AL, and Decatur silt loam (DSL) and was analyzed at the Auburn University Soil Testing Laboratory.

### Pupation success of small hive beetle wandering larvae in Kalmia loamy sand

This experiment determined the optimum success of SHB wandering larvae at five different concentrations in natural non-autoclaved and sterile autoclaved KLS in order to set a control standard for future experiments. A total of 2,250 wandering larvae of the same age and generation were collected from the SHB colony and split equally into three groups. Soil condition consisted of natural non-autoclaved soil and sterilized autoclaved soil which was prepared at 15% moisture by weight (Neumann et al., 2013). Concentrations of 0, 5, 10, 20, and 40 SHB wandering larvae were evaluated. This experiment consisted of two soil conditions X five SHB wandering larvae densities X five replications X three repeated experiments arranged in a randomized complete block design (RCBD). In total, 150 experimental units were evaluated. Soil condition consisted of natural non-autoclaved soil and sterilized autoclaved soil at 15% moisture by weight (Neumann et al., 2013). Concentrations of 0, 5, 10, 20, and 40 SHB wandering larvae were added to 130 cm^3^ of the respective soil and allowed 3 min to burrow. A piece of filter paper was then placed on top of the soil, covered with the Petri dish lid and sealed with parafilm (Ellis et al., 2010). After 24 and 48 hr, respectively, the next sets of 50 petri dishes were prepared. All Petri dishes were placed upside down and stored in an incubator at 25°C, 80% relative humidity (RH), in total darkness for 20 days (Ellis et al., 2010; Neumann et al., 2013). To control the effects of confounding variables, each replication was placed in two stacks separated by soil condition (autoclaved or non-autoclaved) and then a random number generator determined the order each of the five units in both stacks should be placed. All five units in both the autoclaved and non-autoclaved stacks for each replication were placed in the same order. Each replication stack was then spaced evenly throughout the incubator in plastic tubs using a randomized complete block design (RCBD) with each tub containing one block. After 20 days, the contents of each Petri dish were shaken into a bin and each SHB was accounted for. SHB were each documented as a live larva, pupa, or adult, or a dead larva, pupa, or adult. Percent mortality was calculated by dividing the total number of dead larvae, pupae, and adults over the total population of SHB in each dish. Percent success pupation was calculated by dividing the total number of alive adults by the total SHB population in each dish. The SHB concentration that had the most successful emergence percentage was used for the remaining objectives.

### Entomopathogenic nematode efficacy of small hive beetle wandering larvae

This experiment determined optimal efficacy of six commercially purchased EPN species at six population density levels in natural non-autoclaved and sterile autoclaved KLS soil. Commercially available EPN*, Steinernema feltiae, S. riobrave, S. kraussei, S. carpocapsae, H. bacteriophora*, and *H. indica* were purchased to determine which had the best efficacy for SHB. Six population densities of each of the EPN were evaluated and included 0, 121, 243, 485, 971, and 1941 IJ3’s per 1 mL inoculum the soil. These populations were achieved following methods by Ellis et al. 2010 with 1 mL concentrations of 0, 5, 10, 20, 40, and 80 IJ3s per cm^3^ soil. This experiment consisted of six EPN species X six EPN concentrations X two soil conditions X five replications arranged in a RCBD and was repeated twice. Petri dishes with soils were set up as described previously. Five SHB wandering larvae were added per Petri dish as previously determined. Petri dishes inoculated with a population of 0 received 1 mL of water. Soil was inoculated equally in five locations in the Petri dish – the center, 0^o^, 90^o^, 180^o^, and 270^o^ approximately 2.5 cm away from the edge of the Petri dish. Inoculum was added to the soil, filter paper applied, the lid was secured, and placed in the incubator as previously described. This experiment was designed as a split-plot RCBD, with EPN species type as the whole plot, and soil condition as the subplot. After 10 days, SHB were recovered as previously described and dissected under a Stereo microscope at 40x for visual confirmation of nematode parasitism (Ellis et al., 2010).

The second part of this experiment focused on the most promising EPN species and concentrations, and soil condition. Laboratory reared *S. carpocapsae, S. riobrave,* and *H. indica* were tested at concentrations of 0, 10, 40, and 80 IJs per cm^3^ soil in natural non-autoclaved KSL soil. This experiment evaluated three EPN species X four EPN concentrations X five replications arranged in a RCBD and was repeated twice. For moisture content, we added 50% field capacity of KLS to the prepared soil. The moisture content measurement needed to change for this experiment because the final objective used three soils instead of one and moisture added by percent by weight will not provide an equal amount of soil moisture available between soil particles for EPNs to facilitate movement. This test was constructed and incubated and after 10 days SHB were evaluated as previously described.

### Entomopathogenic nematode efficacy of small hive beetle wandering larvae in Kalmia loamy sand, Benndale fine sandy loam, and Decatur silt loam

To determine if soil type changed the efficacy of EPNs on SHwB, three soil types were evaluated; KLS, BFSL, and DSL were selected and placed at 50% field capacity moisture level. Each 25 mm × 100 mm Petri dish contained one of the three soil types, five SHB wandering larvae, and *S. carpocapsae, S. riobrave*, or *H. indica* at concentrations of either 0, 10, or 80 IJ per cm^3^ soil. The experimental unit and method for controlling confounding variables was the same as described previously. This experiment had a split-split-plot within the RCBD where blocks contained nine stacks separated by replication, then soil type, and finally by EPN species. Visual confirmation and documentation of nematode parasitization of each SHB was performed as previously described.

### Statistical analysis

All data were analyzed in SAS software (Version 9.4, SAS Institute, INC, Cary, NC) using PROC GLIMMIX. Response data from repeated tests were combined where no interactions were found between repeated trials. Treatment LS-means were separated by Tukey–Kramer at the significance level of *p*≤0.05. Standard error of the mean (SEM) was calculated for each parameter mean.

## Results

### Pupation success of small hive beetle wandering larvae in Kalmia loamy sand

**Figure 1:**
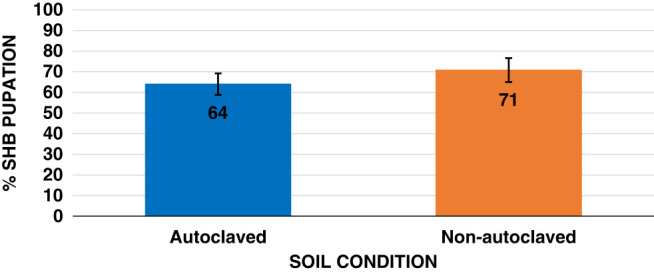
Pupation survival rates of *Aethina tumida,* small hive beetle, wandering larvae in sterile autoclaved or natural non-autoclaved Kalmia loamy sand soil after 20 days. Means of bars with the same letter are not significantly different (Tukey–Kramer, *p* ≤ 0.05).

**Figure 2:**
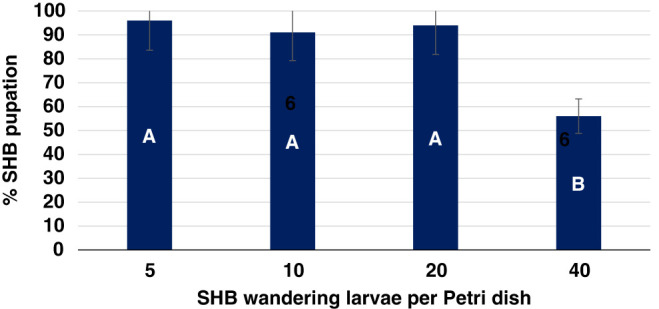
Pupation survival rates of *Aethina tumida,* small hive beetle, wandering larvae at four population densities in sterile autoclaved and natural non-autoclaved Kalmia loamy sand after 20 days. Means of bars with the same letter are not significantly different (Tukey–Kramer, *p*≤0.05).

The interaction between SHB population density, and natural non-autoclaved or autoclaved KLS soil was not significant (*p*  > 0.0993). SHB emergence results indicate that the soil condition of natural or autoclaved did affect pupation significantly. SHB emergence was 64% in autoclaved soil and 71% emergence in the natural non-autoclaved soil (Fig. 1). Successful pupation of the SHB decreased by 41.7% (*p <  *0.001), with overall pupation success ranging from 96.0% at the lowest level of five wandering larvae and 56.0% at the highest level of 40 wandering larvae per Petri dish (Fig. 2). A population density of 5 SHB wandering larvae per petri dish was statistically similar to using population densities of 10 or 20 larvae per petri dish, thus this population levels was utilized in the following experiments.

### Entomopathogenic nematode efficacy of small hive beetle wandering larvae

**Figure 3:**
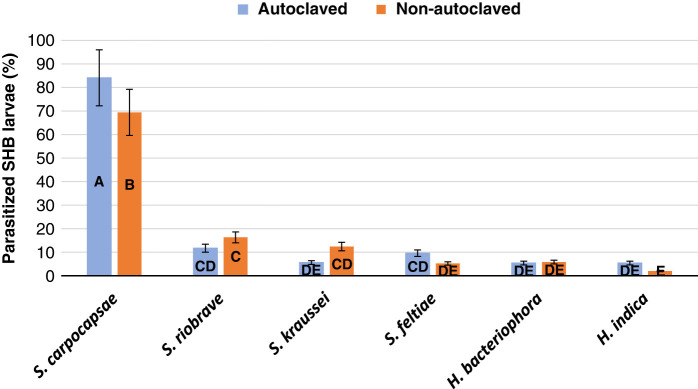
Percent parasitization of *Athina tumida*, small hive beetle, wandering larvae after treatments of six purchased entomopathogenic nematode species in sterile autoclaved or natural non-autoclaved Kalmia loamy sand after 10 days. Means of bars with the same letter are not significantly different (Tukey–Kramer, *p*≤0.05).

**Figure 4:**
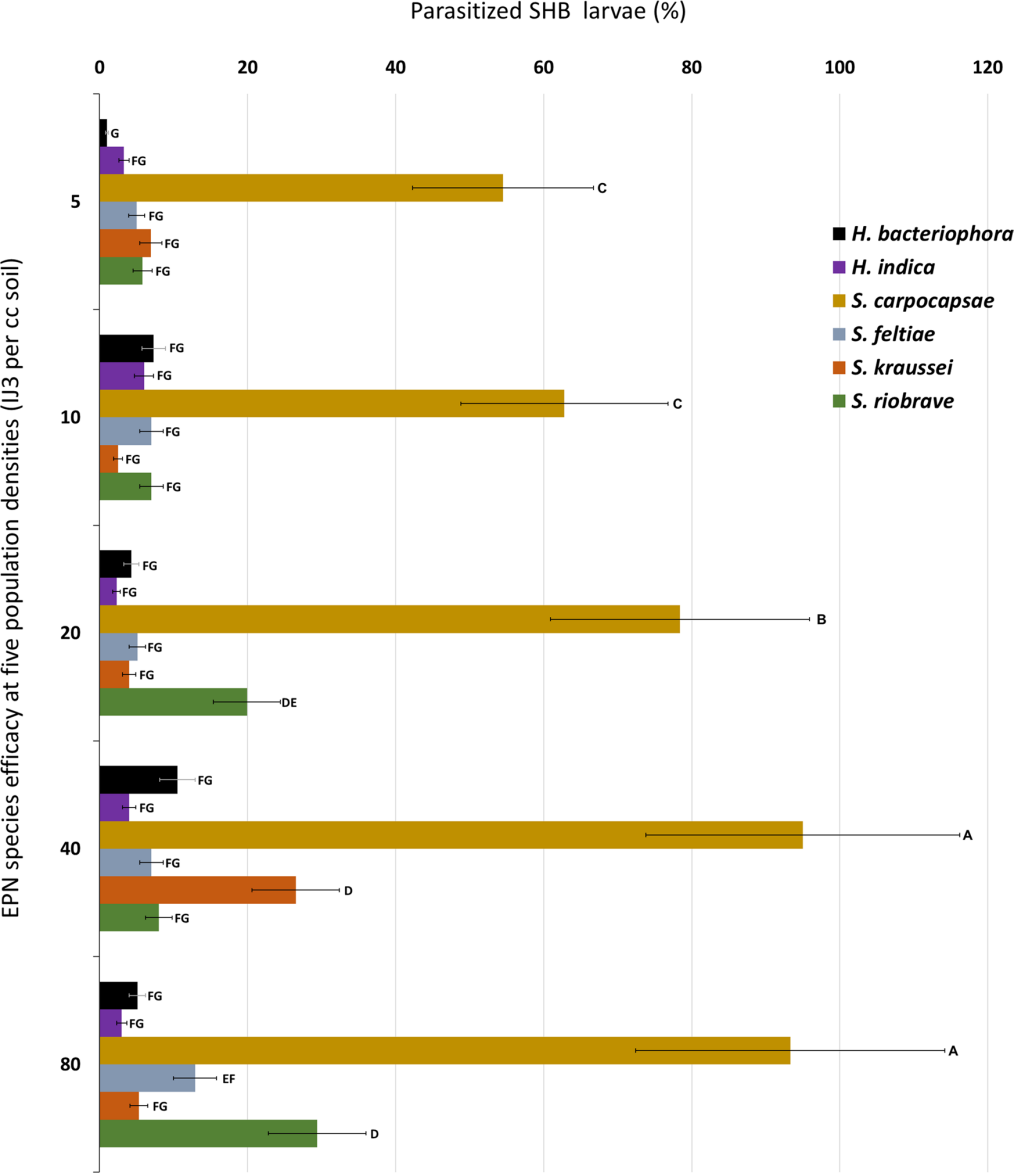
Percent parasitization of *Athina tumida*, small hive beetle, wandering larvae after treatments of six purchased entomopathogenic nematode species at five EPN population densities in sterile autoclaved and natural non-autoclaved Kalmia loamy sand after 10 days. Means of bars with the same letter are not significantly different (Tukey–Kramer, *p*≤0.05).

The test to determine the efficacy of the six EPN species and five EPN populations levels in the autoclaved and non-autoclaved soil indicated no significant interaction between all three factors at *p* > 0.0652. Interactions were observed between EPN species and EPN population densities (*p* < 0.0001), and EPN species and soil treatment (*p < *0.0001). In sterile autoclaved soil, EPN treatment efficacy of all six EPN species individually with combined data for all five population densities ranged from 84.1 to 5.4% (Fig. 3). In natural non-autoclaved soil, EPN treatment efficacy ranged from 69.4 to 2.0%. *Steinernema feltiae*, *S. riobrave*, *S. kraussei*, *H. bacteriophora*, and *H. indica* all obtained statistically similar efficacy, with no significant difference between soil condition (*p* > 0.1887). *Steinernema carpocapsae* obtained higher efficacy than all other species tested in both the sterile autoclaved and natural non-autoclaved soils (*p* < 0.0001) and across all five-population densities (*p*  < 0.0001). At each of the five population densities, parasitization success was significantly different between all six EPN species (*p* < 0.0001). Percent parasitization of SHB wandering larvae at the five EPN population densities varied between 54.5 and 95.0% (Fig. 4). The population density levels of 80 and 40% were effective in parasitizing 94% of the SHB wandering larvae. The 20% population density was effective in parasitizing 78% SHB wandering larvae followed by the 10 and 5% population densities, which were both similar in parasitizing 58% SHB larvae. *Steinernema riobrave* efficacy was highest at 80% population density, *S. kraussei* efficacy was highest at 40% population density, and efficacy for all other EPN species tested was not significantly increased with higher population densities. Based on the results from this experiment, *S. carpocapsae*, *S. riobrave*, and *H. indica* at population densities of 10, 40, and 80%, respectively, were selected for further testing.

**Figure 5:**
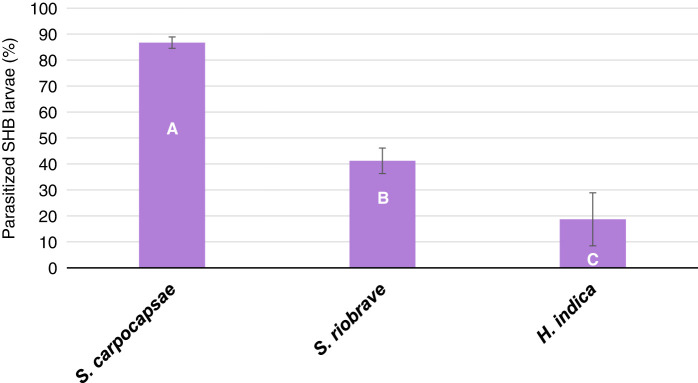
Percent parasitization of *Aethina tumida*, small hive beetle (SHB), wandering larvae after treatments of three USDA reared entomopathogenic nematode (EPN) species in non-autoclaved Kalmia loamy sand at 50% field capacity after 10 days. Means of bars with the same letter are not significantly different (Tukey–Kramer, *p* ≤ 0.05).

**Figure 6:**
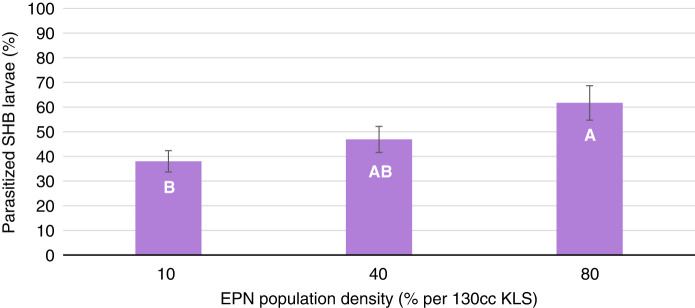
Percent parasitization of *Aethina tumida*, small hive beetle (SHB), wandering larvae after treatments of three USDA reared entomopathogenic nematode (EPN) species at three population densities in non-autoclaved Kalmia loamy sand at 50% field capacity after 10 days. Means of bars with the same letter are not significantly different (Tukey–Kramer, *p* ≤ 0.05).

Further confirming the optimum EPN species and population density, the test observing a two-way interaction between laboratory reared *S. carpocapsae*, *S. riobrave*, and *H. indica*, at three population densities in non-autoclaved KLS found no significant interaction (*p* > 0.4604). Overall parasitization of SHB wandering larvae varied by 78.4% across the three EPN species with *S. carpocapsae* being more efficient at parasitization (Fig. 5). Efficacy of all EPN species increased 38.4% with increasing population density of at 10 to 80% (Fig. 6). The EPN population density with the highest percent efficacy of 61.7% occurring at the highest population density of 80%. Based on the results from this experiment, EPN efficacy in KLS at 50% field capacity is greatest when inoculated at the higher population density of 80%. For this reason, the remaining experiment continued to observe efficacy of *S. carpocapsae*, *S. riobrave*, and *H. indica* at population densities of 10 and 80%.

### Entomopathogenic nematode efficacy of small hive beetle wandering larvae in Kalmia loamy sand, Benndale fine sandy loam, and Decatur silt loam

**Figure 7:**
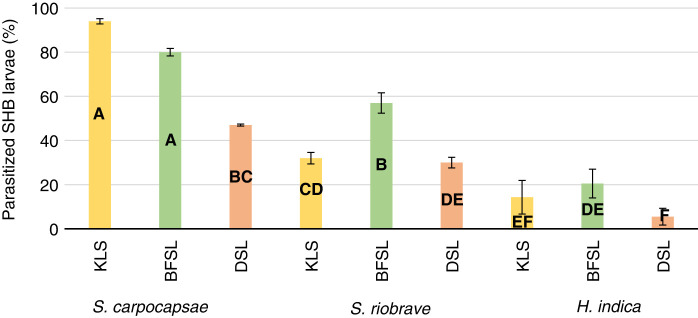
Percent parasitization of *Aethina tumida*, small hive beetle, wandering larvae after treatments of three USDA reared entomopathogenic nematode species in a Kalmia loamy sand, Benndale fine sandy loam, and a Decatur silt loam soils types at 50% field capacity after 10 days. Means of bars with the same letter are not significantly different (Tukey–Kramer, *p* ≤ 0.05).

**Figure 8:**
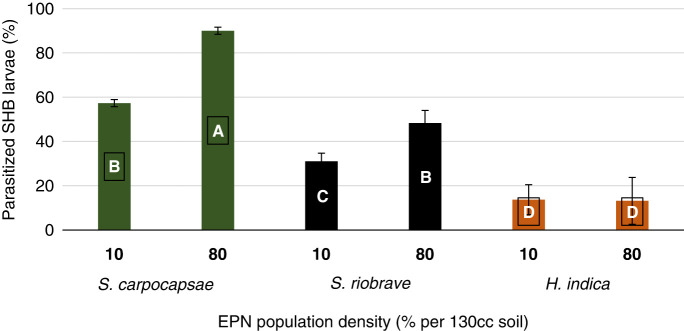
Percent parasitization of *Aethina tumida*, small hive beetle, wandering larvae after treatments of three USDA reared entomopathogenic nematode species at two EPN population densities after 10 days. Means of bars with the same letter are not significantly different (Tukey–Kramer, *p* ≤ 0.05).

The test was expanded to include varying soil types present in Alabama. A three-way interaction test between three EPN species, three soil types, and two EPN population densities showed no significant interaction at (*p* > 0.1930). There was a significant two-way interaction between the three EPN species and the three soil types at (*p* > 0.0003), as well as the three EPN species and two EPN population densities at (*p* < 0.0016). *Steinernema carpocapsae* continued to be the most effective EPN to infect SHB when the soil types were expanded to include the BFSL and DSL soil. Across the varied soil types, *S. carpocapsae* obtained the highest parasitization with 94.0% in KLS, 80.0% BFSL, and 47.0% in DSL (Fig. 7). *Steinernema riobrave* EPN obtained highest parasitization rates of 57.0% in BFSL soil which was 28.8% lower than *S. carpocapsae* in BFSL but the highest efficacy overall for the *S. riobrave* EPN species. *Heterorhabditis indica* EPN had similar parasitism rates in the KLS and BFSL sandy soils and was least parasitic in the DSL clay soil. Efficacy across soil types varied by 84.8% (*p*  <  0.0001) in KLS, 74.4% (*p* < 0.0001) in BFSL, and 85.3% (*p* = 0.0025) in DSL. Population density did affect EPN parasitism when testing the high and low levels. *Steinernema carpocapsae* was most efficacious of the three species and parasitized more SHB larvae at the higher population density of 80% than the lower 10% level (Fig. 8). *Steinernema riobrave* followed a similar pattern to *S. carpocapsae*, parasitizing more SBH at the higher 80% level than the 10%. *Steinernema riobrave* was more parasitic than *H. indica* but was less parasitic than *S. carpocapsae*. *Heterorhabditis indica* was equally pathogenic at both population densities. At the 10 and 80% EPN population density, *S. carpocapsae* obtained the highest percent parasitization of SHB larvae. Parasitization increased from 10% IJ3 population density to 80% IJ3 population density by 35.8% for *S. riobrave* and 57.1% for *S. carpocapsae.* Parasitization decreased by 3.6% for *H. indica* from the 10% IJ3 population density to 80% IJ3 population density. *Steinernema carpocapsae* obtained the highest percent parasitization across all three soils and at both population densities.

## Discussion

Biological control agents that can control a pest of *A. mellifera* colonies while not harming *A. mellifera* individuals are important to consider as a part of an integrated pest management program for control of SHB. Based on previous literature, all six EPN species used in this study had potential as biological control agents for SHB wandering larvae in Europe or North America (Cabanillas and Elzen, 2006; Cuthbertson et al., 2012; Ellis et al., 2010; Hill et al., 2016; Shapiro-Ilan et al., 2010). These previous studies were mainly conducted using sterilized sand and various EPN inoculation methods. Many also standardized soil moisture levels as a percentage of water based on weight of the soil. This method of calculating soil moisture does not translate to various natural soil types. In our study, we confirmed EPN efficacy on SHB wandering larvae in various Alabama soil types using field capacity to standardize low soil moisture conditions and bridge the gap between laboratory bioassays and future field bioassays.

Results of the first experiment observing survival rates of SHB wandering larvae at various population densities supported the use of five larvae per Petri dish. Similar studies also used five larvae per dish, however their Petri dish sizes were ~154 cm^2^ smaller than what we used (Ellis et al., 2010; Vega et al., 1994). Our results concluded that lower SHB population densities do not significantly impact SHB survival rates, therefore, the use of five SHB larvae per Petri dish is adequate. Consequently, this allowed us to optimize experimental units and replications as less resources were utilized. Small hive beetle wandering larvae are not known to pupate in congregations, may travel away from the soil directly beneath a hive in search for a suitable pupation location, and generally pupate in the top 20 cm of soil (Frake and Tubbs, 2009; Neumann et al. 2013). For this reason, we used larger Petri dish to better simulate SHB dispersal observed in the field. Based on our results and previous findings, five SHB larvae should be an adequate population density in experiments conducted using materials with an internal space between 42.2 and 196.3 cm^3^.

Efficacy varied between the six EPN species and population densities in the initial tests using KLS. Of the six EPN species we tested, only *S. carpocapsae* at an application rate of 80% IJ3 per Petri dish (~1,941 IJ3 per 1 mL inoculum) showed promise as a biological control agent. Cuthbertson et al. (2012) observed the effects of dipping SHB wandering larvae directly into solutions containing *S. carpocapsae*, *S. kraussei*, and *S. feltia* IJ3, treating sand with EPN solutions before adding SHB wandering larvae, and the effects of subsequential applications of EPNs to sand over time. They found similar success as our studies with *S. carpocapsae* across all three techniques. Interestingly, *S. kraussei* and *S. feltiae* were ineffective when SHB wandering larvae were directly exposed to them, however, *S. kraussei* achieved 100% efficacy when applied to sand and allowed to soak into the sand before SHB wandering larvae were added (Cuthbertson et al., 2012). This suggests that EPNs efficacy may also depend on inoculation method.

The two species of *Heterorhabditis* we tested showed less than 50% parasitization of SHB wandering larvae in every soil type and at every population density they were tested in. Previous bioassays conducted using *H. bacteriophora* to control SHB wandering larvae in autoclaved soil in Florida showed a lower rate of parasitism which was similar to our results (Ellis et al., 2010). Previous bioassays conducted with *H. indica*, however, had almost 100% efficacy between 9 days and 14 weeks post-inoculation (Ellis et al., 2010). Another study conducted with *H. indica* also indicated high efficacy of SHB wandering larvae over 10 to 15 days (Shapiro-Ilan et al., 2010). The main difference between our experiments and these two studies appears to be the method of inoculation and longevity of *H. indica* in soil post-inoculation. In both studies mentioned, *H. indica* performed best when inoculated via an infected cadaver instead of an aqueous solution. At this time there are no field bioassays involving inoculation of *H. indica* infected cadavers in the soil under *A. mellifera* hives. Variability of efficacy between experiments conducted using purchased EPNs verses USDA reared EPNs could be caused by factors such as nematode age at time of inoculation and rearing conditions and methods. Purchased EPN species arrive as a mixture of all juvenile stages and are reared by of third-party laboratories that may use different rearing conditions and methods. EPN species reared by the USDA were the same age at time of inoculation, IJ3, and experienced the same rearing conditions and methods.

Efficacy varied between *S. carpocapsae*, *S. riobrave*, and *H. indica* in KLS, BFSL, and DSL soils. Efficacy of *S. carpocapsae* and *S. riobrave* appears to be directly related to increase in population density at time of inoculation. *Steinernema carpocapsae* appeared to be the most effective at all population treatment levels. The success of *S. carpocapsae* as a biological control agent for SHB wandering larvae are similar to results found by Cabanillas and Elzen (2006), Cuthbertson et al. (2012), Shapiro-Ilan et al. (2010). *Steinernema carpocapsae* was also the most effective in all three soil types tested. This suggests that *S. carpocapsae* performs better than *S. riobrave* and *H. indica* under low moisture conditions in the loamy sand, sandy loam, and silt loam found in Alabama. The ability of *S. carpocapsae* to parasitize SHB wandering larvae in low moisture conditions is supported by Grant and Villani (2003), Koppenhöfer et al. (1995), Kung et al. (1991). Previous studies noted that *S. carpocapsae* can survive for up to 16 weeks in sand, sandy loam, clay loam, and clay (Kung et al., 1990a) and prefers to hunt near the soils surface (Divya and Sakar, 2009; Moyle and Kaya, 1981). All three of these characteristics further support the idea that *S. carpocapsae* is a viable biological control agent in Alabama.

In summary, of all six EPNs tested, *S. carpocapsae* had the highest infection rates above 80% after 10 days in three soil types at 50% field capacity. Results confirmed that EPNs efficacy significantly differ based on soil texture and composition. *Steinernema carpocapsae* and *S. riobrave* were better able to control SHB at the higher population density levels. Results suggest that *S. carpocapsae*, inoculated at  > 80% IJ3s per cm^2^ soil, is a promising biological control agent for beekeepers in Alabama with hives on loamy sand, fine sandy loam, or silt loam soils during times of low moisture, which is common in this region. One way for beekeepers in Alabama to determine when their county is experiencing low moisture conditions is through the National Integrated Drought Information System (Hartman, 2020, https://www.drought.gov/drought/rcc/southeast). The EPN biological control agent, *S. carpocapsae* has good potential to effectively manage SHB when added to a bee keeping integrated pest management program.
